# Supplementary material for: Distinct Montages of Slow Oscillatory Transcranial Direct Current Stimulation (so-tDCS) Constitute Different Mechanisms during Quiet Wakefulness
Source: Brain Sci. 2019 Nov 14;9(11):324. doi: 10.3390/brainsci9110324 (PMC6896026; doi:10.3390/brainsci9110324)
Supplement: Supplementary file 1 [file brainsci-09-00324-s001.pdf]

Supplementary Note 1. Supplementary experiment with Group Small

We were able to acquire a subgroup of subjects from Group Small for two additional sessions; the procedure was identical to the main experiment, except that as stimulation frequency individual alpha frequency instead of the slow oscillation frequency was used. Nine out of 16 subjects participated in the supplementary experiment and received monetary compensation. Individual alpha frequency within 7-14 Hz (mean frequency = 9 Hz ± 0.6) was obtained from the average baseline data (prior to stimulation) and the five stimulation-free intervals during the sham session of the main experiment. Performance on the finger sequence tapping task was not altered by IAF-tDCS as compared to sham (COND x TIME: $F(1, 8) = 2.75$, $p = 0.14$).

Supplementary Note 2. No change of delta and spindle power related to sleepiness scale.

Due to the significant difference in sleepiness scale found in Group Large, power of rhythms characteristic for sleep were additionally investigated, i.e. slow wave activity (0.5-4 Hz) and sleep spindles (12-15 Hz) in three-way rmANOVAs. There was no significant main effect of COND for either rhythm ($p > 0.10$).

Supplementary Note 3. Overview of ANOVA results.

Table 1

Test	Effect	Group Small		Group Large	
		F value	P value	F value	P value
SO	Cond	0.97	0.34	0.04	0.85
	Time	1.24	0.30	0.67	0.53
	Cond x Topo	1.02	0.37	0.38	0.75
	Cond x Time	1.26	0.29	1.35	0.27
Theta	Cond	0.01	0.92	3.59	0.08
	Time	0.66	0.54	0.67	0.59
	Cond x Topo	0.69	0.54	1.29	0.28
	Cond x Time	1.70	0.20	0.31	0.84
Alpha	Cond	5.56	*0.03**	0.17	0.69
	Time	2.71	0.07	2.31	0.09
	Cond x Topo	4.68	*0.04**	0.34	0.62
	Cond x Time	0.55	0.61	0.63	0.61
IAF	Cond	0.20	0.66	6.26	*0.02**
	Time	2.59	0.08	2.86	0.05
	Cond x Topo	0.30	0.64	5.96	*0.01**
	Cond x Time	0.69	0.52	0.56	0.63
θ_S α_{DS}	Cond	0.02	0.90	0.23	0.64
	Cond x Topo	0.55	0.71	2.78	*0.03**

Table 1. Overview of F statistics for relevant Condition and interactions with Condition of EEG data. All values were rounded up to two decimal points. Asterisks represent $p < 0.05$; Greenhouse-Geisser corrections were applied if necessary. Degrees of freedom, Cond: $F(1,15)$; Time: $F(5,75)$; Cond x Topo interaction: $F(11,165)$, Cond x Time interaction: $F(5, 75)$.

Table 2

Test	Effect	Small		Group Large	
		F value	P value	F value	P value
Positive	Cond	0.01	0.94	0.37	0.56
	Cond x Time	6.86	*0.02**	0.14	0.71
Negative	Cond	3.92	0.07	2.91	0.11
	Cond x Time	2.76	0.12	0.26	0.62
SSS	Cond	0.63	0.44	5.00	*0.04**
	Cond x Time	10.08	*0.006**	0.65	0.43
FSTT	Cond	0.06	0.81	0.50	0.49
	Cond x Time	5.69	*0.03**	0.24	0.63

Table 2. Overview of F statistics for relevant Condition and interactions with Condition of behavioral and control data. All values were rounded up to two decimal points. Asterisks represent $p < 0.05$; Greenhouse-Geisser corrections were applied if necessary. Degress of freedom, Cond: $F(1,15)$; Cond x Time interaction: $F(1,15)$.
